# Assessment of new composites containing polyamide-6 and lead monoxide as shields against ionizing photonic radiation based on computational and experimental methods

**DOI:** 10.1038/s41598-022-13556-9

**Published:** 2022-06-03

**Authors:** Shahryar Malekie, Hassan Shooli, Mohammad Amin Hosseini

**Affiliations:** 1 Radiation Application Research School, Nuclear Science and Technology Research Institute, PO Box 31485-498, Karaj, Iran; 2 Department of Nuclear Engineering, Islamic Azad University Arsanjan Branch, Arsanjan, Iran; 3 Ionizing and Non-Ionizing Radiation Protection Research Center (INIRPRC), School of Paramedical Sciences, Shiraz University of Medical Sciences, Shiraz, Iran

**Keywords:** Materials science, Nanoscience and technology

## Abstract

This study aimed to introduce new composites, containing polyamide-6 (PA6) and lead monoxide (PbO), to protect against ionizing photon sources used for diagnostic and therapeutic purposes. Five composites, containing various weight percentages of PbO filler (0, 5, 10, 20, and 50%), were developed in this study. Initially, the numerical attenuation value was estimated using the XMuDat program by calculating the mass attenuation coefficients at different energy levels. Next, the samples were synthesized based on the melt-mixing method in a laboratory mixing extruder. Their characteristics were also determined by scanning electron microscopy (SEM), energy dispersive X-ray (EDX) analysis, X-ray diffraction (XRD), and thermogravimetric analysis (TGA). Finally, experimental radiation attenuation tests were carried out. Based on the SEM results, the acceptable filler weight percentage was up to 20%. However, substantial aggregates were formed at the highest weight percentage. The results of XRD analysis showed a higher tendency for crystallization by decreasing the amorphous area while increasing the filler weight percentage. Moreover, the mass loss rate was monitored at different temperatures, revealing that the filler incorporation improved the thermal durability of the samples. The radiation results showed a good agreement between the experimental and computational data, except when aggregates formation was substantial. The experimental data revealed that when the lead weight percentage increased from 0% (crude PA6) to 50%, the half-value layer decreased from 3.13 to 0.17 cm at an energy level of 59 keV and from 7.28 to 4.97 cm at an energy level of 662 keV. Following the studied mechanism, the superiority of lead/polyamide composites can be found in the high adsorption of photon radiation at low energies (E < 0.20 MeV) and significant attenuation at medium and higher energies. Considering these promising results, the shielding properties of these composites can be further analyzed via more practical investigations.

## Introduction

Today, the risk of intended or unintended exposure to ionizing radiation has increased in humans due to their increasing tendency to develop and use new technologies^1,2^. The exposure to ionizing radiation may be caused by cosmic rays, fuel processing, nuclear fusion processes, industrial radiation processing (especially in the food and health sectors), and long-term use of X-rays and gamma rays for diagnostic and therapeutic purposes^3,4^. Despite the advantages of ionizing radiation, radiation hazards and protection remain serious health concerns^3,5^.

The use of adsorbents for the development of protectants is a well-known strategy. Lead, as an element best known for its high atomic number, shows high efficiency in absorbing radiation. Besides, due to its high abundance and cost-effectiveness, major attention has been paid to this element. For many years, lead products have been considered the best available option for fabricating fixed (e.g., walls and blocks) or portable (e.g., aprons, glasses, gloves) protection equipment^6^. However, the challenges and problems of using lead products have become more prominent over time. Some studies have addressed the disadvantages of using bulk lead in the construction of shields such as the high weight of lead products, health and environmental challenges due to high lead toxicity, and high fragility of these products^7,8^.

In recent years, different strategies have been employed to obviate the mentioned challenges. One of the well-known strategies is the use of materials such as bismuth, tungsten, and tin as alternatives to lead^7–12^. Furthermore, the application of a nanocomposites as a novel approach can resolve many of these challenges. In this approach, polymers (matrix) and nanoscale heavy metals (fillers) are often used. It is assumed that with a suitable distribution of particles in the matrix, the surface -to-volume ratio increases, leading to a higher radiation absorption efficiency and an optimal reduction in the product size^8,13^. Therefore, the radiation protection products are expected to have higher efficiency, flexibility, chemical and thermal stability, and biocompatibility^6,8^.

To overcome the disadvantages associated with using lead, previous studies have come up with promising outcomes by using nanosize or microscale lead (rather than bulk use) with various chemical structures (e.g., PbO, PbO_2_, Pb_3_O_4_, or chloride forms). Overall, recent research findings enable optimizing the use of lead in new forms^6^. So far, numerous experimental studies have assessed the applicability of lead polymers for radiation protection. Thus, they have analyzed different concentrations of lead monoxide (PbO) in unsaturated polyesters with a ‍cesium-137 source^14^ by adding modified nanoclay^15^.

Satisfactory radiation protection efficiency and structural properties have been reported for nanoscale PbO compared to bulk PbO in heavy polyethylene^16^ or a linear low-density polyethylene when exposed to mid- and high-energy gamma radiation^17^. Other compounds such as nano-PbO reinforced into epoxy resins^18^, polyvinyl alcohol^19^, and polyesters^20^ have also demonstrated desirable radiation protection features. In line with experimental studies, several computational studies have confirmed the efficiency of lead-based composites^21,22^. Moreover, polymer-based composites and nanostructures have shown unique features in other radiation fields, including the basic design and development of ionizing radiation dosimeters^23–30^. Of the existing polymers, polyamide 6 is also known as a fiber-forming polymer with unique thermal, mechanical, and chemical properties. It has recently come in widespread use for research and development on composite materials, especially textile fibers^31^.

Despite the reported benefits of polymeric composites in ionizing radiation and the possibility of reusing optimized lead composites as radioprotective materials, there is a need for further scientific research on these composites. Therefore, in the present study, new composites were fabricated with PbO and polyamide-6 (PA6). Furthermore, the properties of these composites to protect against X-rays and gamma rays were investigated in a broad energy range using computational and experimental methods. To the best of our knowledge, this is one of the first reports on composites containing lead fillers in a polymeric PA6 matrix.

## Materials and methods

### Theoretical section: estimation of radiation attenuation properties of the compounds

This study primarily aimed to assess the protective effects of novel PA6/PbO composites against X-ray and gamma radiation using computational approximation and experimental methods. The studied composites contained PbO (ρ = 9.53 g/cm^3^) as the filler and PA6 ([C_12_H_22_N_2_O_2_]_n_, ρ = 1.13 g/cm^3^) as the polymeric matrix^32^. In this study, five composites containing different weight percentages of PbO (0, 5, 10, 20, and 50%) were fabricated. Table 1 shows the distribution of elements in each compound. The nominal density of each compound was calculated based on the American Society for Testing and Materials (ASTM) D792-91 standard (1991) (the last column in Table 1), using the following equation^33^:

1 $$\rho =\frac{1}{\frac{{w}_{1}}{{\rho }_{1}}+\frac{{w}_{2}}{{\rho }_{2}}}$$

where w1 and ρ1 denote the weight percentage and density of the fillers (i.e., PbO), respectively, and w2 and ρ2 represent the corresponding parameters for the matrix (i.e., PA6), respectively.

**Table 1:** Characteristics of the studied composites with five different PA6 and PbO combinations.

Sample	wt% (PbO)	H	C	O	N	Pb	ρ_total_ (g/cm^3^)
PA6 (crude)	0	0.098	0.637	0.124	0.141	0.000	1.130
PA6/PbO-5%	5	0.093	0.605	0.118	0.138	0.046	1.182
PA6/PbO-10%	10	0.088	0.573	0.111	0.134	0.093	1.239
PA6/PbO-20%	20	0.078	0.509	0.099	0.127	0.186	1.372
PA6/PbO-50%	50	0.064	0.417	0.081	0.117	0.320	2.020

Moreover, it is possible to approximate the radiation absorption and attenuation properties of materials and compounds both before and after synthesis based on calculations using the dedicated codes and programs. Besides, experimental analysis of attenuation and absorption properties in a wide range of beam energies, which is by itself an interesting research topic, has limitations in practice. Therefore, use of dedicated software programs, followed by validation of the results using experimental tests in exposure to several radioactive sources (i.e., comparison of the results of experimental and computational methods), has always been interesting to researchers^34–37^. Some important parameters for estimating the behavior of materials in exposure to X-rays and gamma rays include the linear attenuation coefficient (μ_L_), total mass attenuation coefficient (μ/ρ), and half-value layer (HVL), which are calculated with dedicated Monte Carlo codes and software programs. One of the well-known dedicated programs is XMuDat^38^, which can determine parameters, such as the mass attenuation coefficient, for six different compounds simultaneously in a broad photon energy range (0.001–50 MeV). In this program, it is essential to include the content of each compound (e.g., type of elements and weight percentages). Calculations are performed according to the algorithms in the program and the data from a reference library. It is possible to use one of the two references by Hubbell et al.^39^ or Boone et al.^40^ as default calculations, where the mass attenuation coefficients of different elements (Z = 1–92) at different energy levels are described. The total mass attenuation coefficient (μ/ρ_total_) of every compound at any given energy level was calculated using the following formula:

2 $$({\frac{\mu }{\rho })}_{total}=\sum {w}_{i}\times ({\frac{\mu }{\rho })}_{i}$$

where *w*_*i*_ is the weight fraction of each element in the compound, and $({\frac{\mu }{\rho })}_{i}$ represents the total mass attenuation coefficient (obtained by dividing the linear attenuation coefficient [μ_L_] by the element density [ρ] at the designated energy level)^41^. It is possible to obtain these numerical values and standard graphs in the software for a maximum of six compounds. By determining the final $({\frac{\mu }{\rho })}_{total}$ value at the specified energy level and considering the density of the composite, the HVL value was determined as follows^6,7^:

3 $$HVL=\frac{ln2}{{\mu }_{L}}$$

In the present study, the numerical values for the total mass attenuation of the five samples listed in Table 1, were calculated in the XMuDat. The input information for the program included data, such as the type and weight fraction of the elements (Table 1). Calculations were performed based on the data library reported by Boone et al. for photon emissions in an energy range of 0.001–10 MeV.

### Experimental section

#### Materials and equipment for characterization of the samples

Polyamide 6 (PA6)-Akulon® F223-D (DSM products) was purchased from the market. Nanoscale PbO powder (in form of a 25-g stock solution) was purchased from Aria Chemical Co. (Tehran, Iran) and used in the experiments after its qualification was verified by X-ray diffraction (XRD) and scanning electron microscopy (SEM) examinations. The following characterization tests were carried out on the PA6/PbO composites: SEM (TESCAN MIRA3, Czech Republic); thermogravimetric analysis (TGA) over a temperature range of 20–600 °C at a rate of 10 °C/min in the air using a TGA analyzer (Netzsch TG 209F3 Tarsus, Frankfurt, Germany) and XRD analysis for phase detection (X'Pert PRO MPD, PANalytical, Almelo, Netherlands). All analyses were performed at room temperature. The X-ray radiation parameters were set at 40 kV and 30 mA (target = Cu Kα, λ = 1.54 Å). It should be noted that in this system, the scans were acquired over a 2θ angle range of 1° to 100° (with 0.026° intervals) in a counting time of 0.5 s. All three mentioned examinations were performed at Razi Metallurgical Research Center (Alborz, Iran) as a service provider.

#### Fabrication of PA6/PbO composites

Five PA6/PbO composites were fabricated at different weight percentages of PbO filler (0, 5, 10, 20, and 50%), using the melt-mixing method. The final fabrication and preparation phases were conducted, using hot and cold pressing processes. During the synthesis process, PA6 and PbO powders were initially weighed, based on the predetermined mass ratios on a four-point digital scale (CAS model) and then placed in an oven at 90 °C for several hours to ensure dehumidification. Before synthesis, granulated PA6 particles were exposed to liquid nitrogen for ten minutes to reaffirm their dryness and fragility. Next, an ultra-centrifugal mill (ZM 200, Retsch, Germany) was used at 14,000 rpm to reduce the particle size to 50 μm.

Considering the melting point of polyamide, the samples were synthesized in a laboratory mixing extruder (Dynisco, USA) over a temperature range of 220–245 °C at 50 rpm. The synthesis conditions were set based on the operator’s experience and a previous study^16^. Finally, the materials were formed into galvanized sheet molds with dimensions of 8 × 8 × 0.1 cm^3^. For molding, the synthesized materials were initially poured into molds and then formed by hot and cold pressing machines (Toyo Seiki Co., Japan). For this purpose, first, hot pressing was performed for each sample at an approximate temperature of 240 °C, considering the melting point of polyamide, less than 25 kg/cm^2^ pressure for five minutes. Next, a cold pressing machine, the plates of which were being cooled by water spray, was applied immediately at a similar pressure for three minutes.

#### Experimental analysis of radiation attenuation

**Figure 1:**
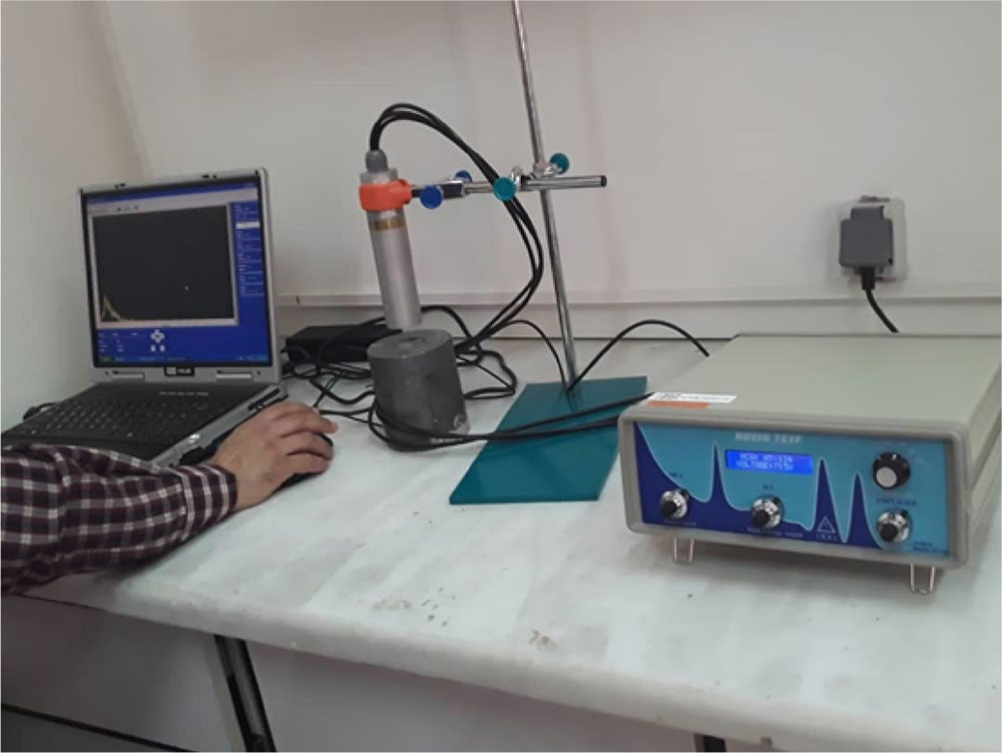
The geometric arrangement used to experimentally evaluate attenuation in the samples. The lead cylinder for narrowing the beams, the detector probe, the power supply, and the spectrum measured by the software are shown in this figure.

The linear attenuation coefficients (μ_L_) of the samples were measured, using an experimental method according to a previous report^10^ over a descending photon energy range with a narrow-parallel beam geometry. After the synthesis and molding of the fabricated samples, they were subjected to experimental radiation tests. First, the sheet composites, with dimensions of 8 × 8 × 0.1 cm^3^, were cut into four sections. To determine the experimental linear attenuation coefficients and HVL values of the composites, a narrow-parallel geometry was considered (Fig. 1) in the presence of two standard low-energy (^241^Am, Eγ = 0.059 MeV) and medium-energy (^137^Cs, Eγ = 0.662 MeV) gamma-ray sources.

The experiments were performed in a specialized laboratory for Secondary Standard Dosimetry Laboratory (SSDL) in Karaj, Iran. The experimental radiation tests (Fig. 1) were performed using a calibrated detector equipped with a CsI(Tl) cylindrical probe (NT-812). The instrument was connected to a power supply and an amplifier (Novin-Teyf, Iran), as shown in Fig. 1. For the initial narrowing of beams, a hole was created in parallel with the probe axis on the lid of the cylindrical chamber, where the standard radiation source was placed (Fig. 1).

**Figure 2:**
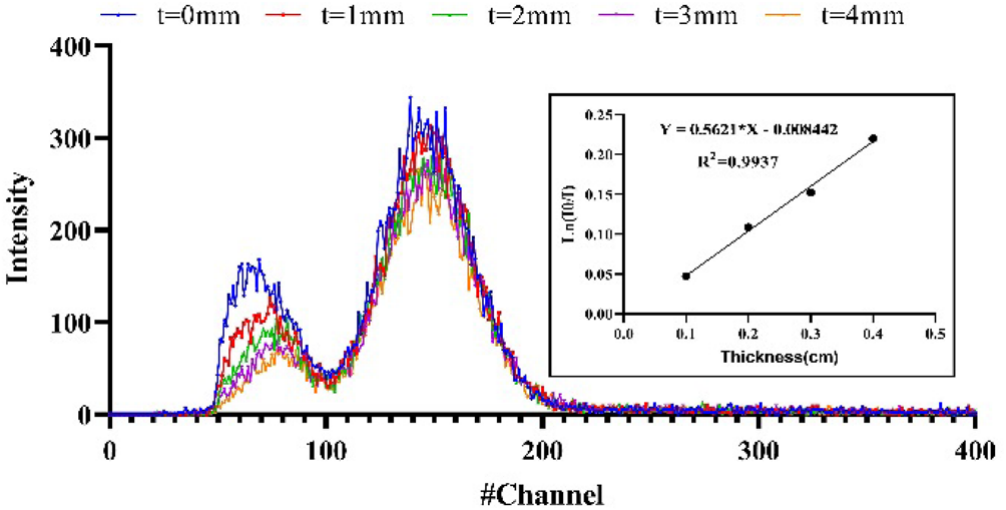
The output curves of the detector system in the presence of an Americium (Am-241) s*ource. The first peak represents the Compton scattering and the second peak (located between channels 100 and 200) displays the main curve for measuring the radiation intensity. The diagram shows how linear regression was used to calculate the linear attenuation coefficients.

To measure the radiation intensity at the desired energy level, the output spectrum of the detector was initially measured in the presence of the test material with different thicknesses (t = 1–4 mm) or in its absence (t = 0). Next, the areas under the curve at the specified peaks (Fig. 2), corresponding to the measured energy intensities (I_0_, I_1_, I_2_, I_3_, and I_4_) in different layers, were used to determine the linear attenuation coefficients, based on the Beer-Lambert formula^10^:

4 $${\mu }_{L}=\frac{\mathrm{ln}\left(\frac{{I}_{0}}{I}\right)}{t}$$

The net value of the linear attenuation coefficient for each sample at a specific energy level was determined by analyzing the linear regression, as shown in Fig. 2. As can be seen, the curve slope corresponds to the linear attenuation coefficient. After determining the experimental value of the linear attenuation coefficient, the HVL value was calculated at each energy level using Eq. (3).

Finally, the estimated linear attenuation coefficients were statistically analyzed by calculating the standard deviation of the linear attenuation coefficients (∆μ_L_) using the following equation^10,42^:

5 $$ \Delta \mu_{L} = \frac{1}{t}\sqrt {\left( {\frac{{\Delta I_{0} }}{{I_{0} }}} \right)^{2} + \left( {\frac{\Delta I}{I}} \right)^{2} + \left( {Ln\frac{{I_{0} }}{I}} \right)^{2} \left( {\frac{\Delta t}{t}} \right)^{2} } $$

It should be noted that the quantities I, I_0_, and t in the above equation are the same as the terms defined in Eq. (4).

## Results and discussion

### Morphological analysis of the samples by SEM

**Figure 3:**
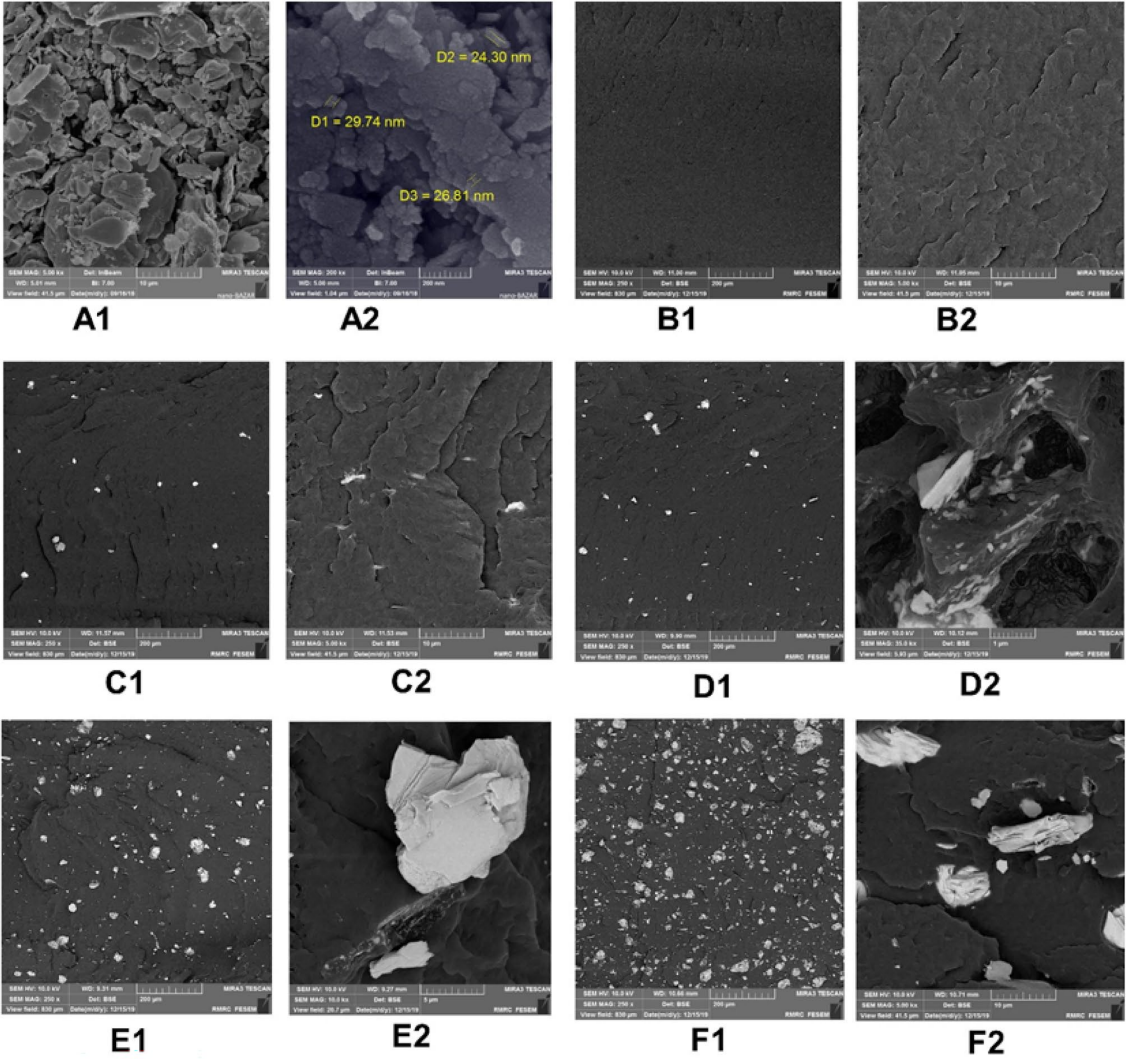
The SEM images of the samples at different magnifications: (**A1,A2**) PbO, (**B1,B2**) PA6, (**C1,C2**) PA6/PbO-5%, **(D1,D2**) PA6/PbO-10%, (**E1,E2**) PA6/PbO-20%, and (**F1,F2**) PA6/PbO-50%.

The pre-synthesis SEM micrographs of PbO powder, samples containing 5, 10, 20 and 50 wt% of PbO, and lead-free samples (crude PA6) are shown in Fig. 3. To better understand the distribution of particles, the images were acquired at two different magnifications. Figure 3A (A1, A2) shows the PbO powder before synthesis with different particle sizes (reaching even < 100 nm). Figure 3B (B1, B2) presents the crude PA6 (filler matrix), allowing for a better assessment of the filler distribution; it seems that the polyamide matrices are arranged as sheets next to each other, similar to microfibers.

Moreover, the filler distribution at weight percentages of 5% and 10% in PA6 after the hot and cold pressing processes is presented in Fig. 3C (C1, C2) and Fig. 3D (D1, D2) respectively. The nano-PbO particles showed a relatively consistent distribution on the polymer surface. However, a slight increase was observed in the filler size, using 10 wt% filler due to the accumulation of particles. Likewise, by increasing the filler weight percentage to 20% and 50%, the asymmetrical increase in the filler size became more noticeable on the surface (E1, E2 & F1, F2); this increase was even more noticeable than that observed in fillers with lower weight percentages.

Despite the accumulation of filler particles in both 20% and 50% PbO composites, 20% PbO seemed to have a more symmetrical distribution on the polymer surface. However, in the 50% PbO sample, an increase in the filler percentage and high adhesion between the filler particles resulted in the accumulation of particles with asymmetrical sizes. In other words, as shown in Fig. 3F (F1, F2), the agglomerations of the fillers were more significant at this concentration, reflecting the formation of stronger van der Waals forces among filler particles, with an increase in the weight percentage of heavy metal fillers (e.g., lead and bismuth) dominating the filler–polymer bonds^10,16,43^. This finding is consistent with the previous studies on composites, containing lead at a weight percentage of 20% or higher^16,17^.

**Figure 4:**
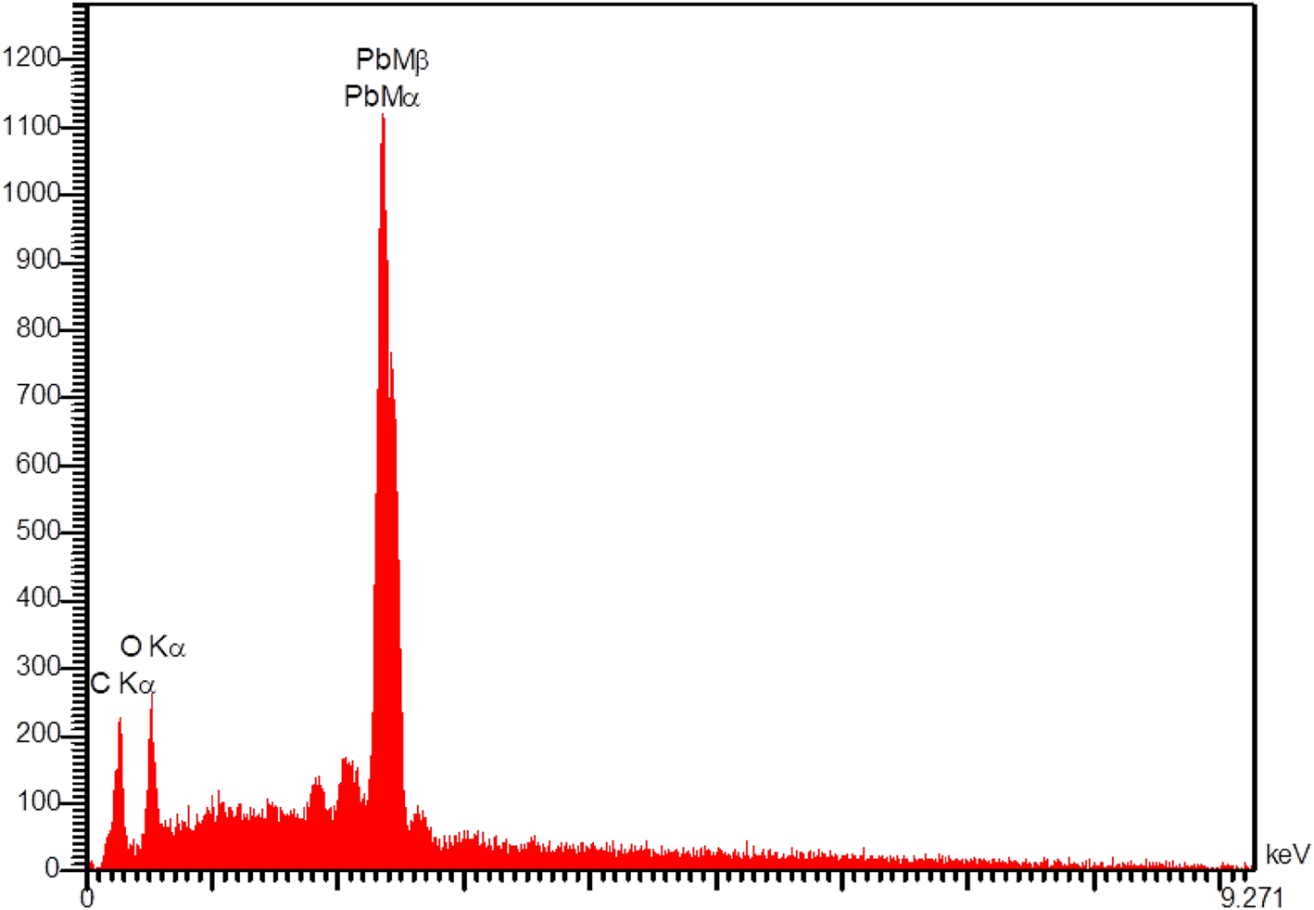
The EDX spectrum of PA6/PbO-10% composite.

To assess the quality of the elements on the surface of the composites, an energy-dispersive X-ray (EDX) analysis was performed on the sample containing 10% lead (Fig. 4). As can be seen, the absorption edges of lead (Mα and Mβ) and other elements, such as carbon and oxygen (Kα), emerged as fingerprints within the selected energy range, confirming the presence of lead (as filler) in the synthesized samples. It should be noted that the absence of hydrogen, as a light element, is related to the inherent limitations of this analytical test^44^.

### Results of XRD analysis of PbO-containing composites

**Figure 5:**
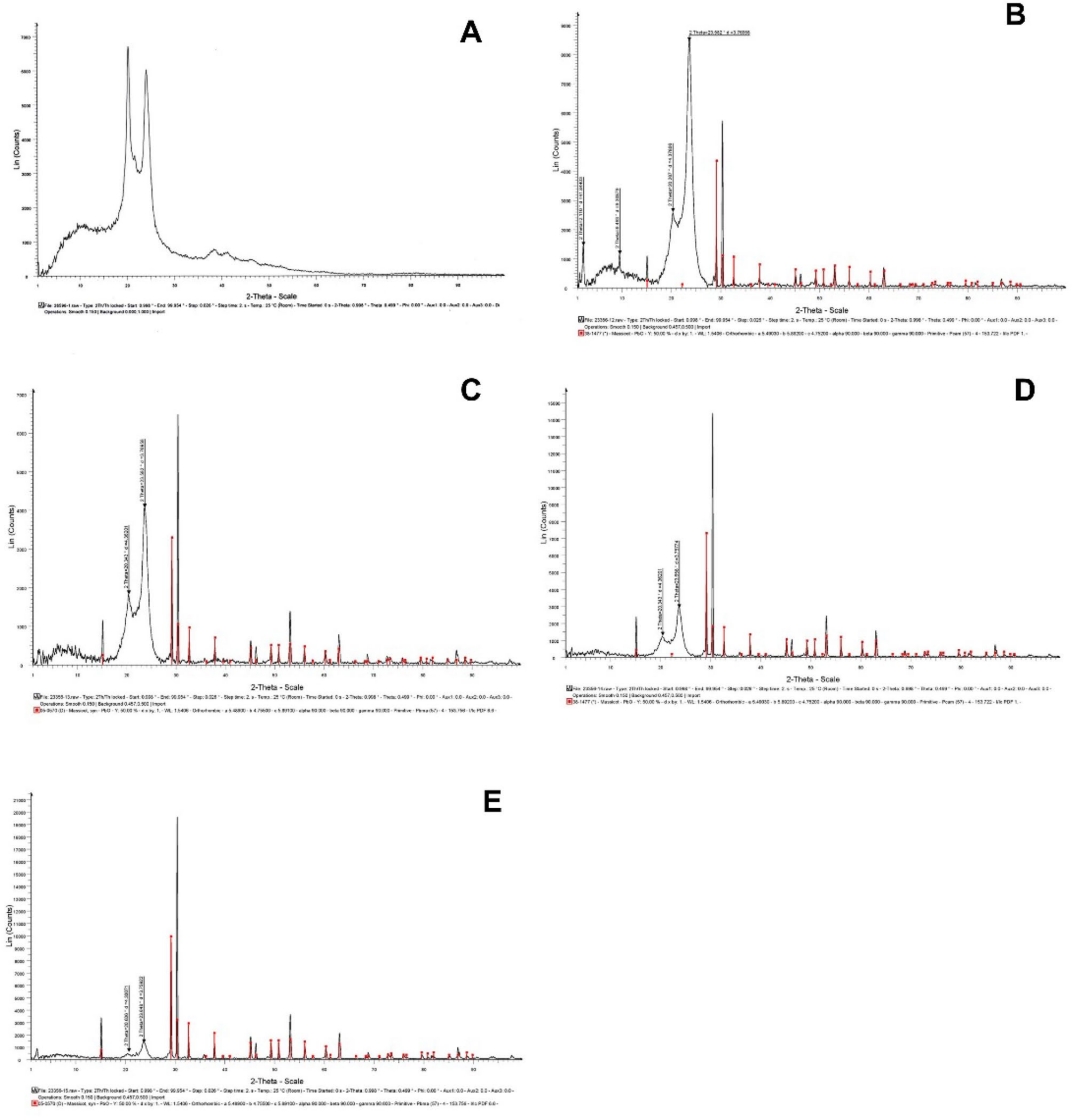
The XRD graphs of the PA6/PbO composites at various concentrations of the fillers, (**A**) 0 wt% (pure PA6), (**B**) 5 wt%, (**C**) 10 wt%, (**D**) 20 wt%, and (**E**) 50 wt%.

Figure 5 presents the results of XRD analysis of the studied composites containing different filler weight percentages (5, 10, 20, 30, and 50 wt%) in the PA matrix at different intensities over different 2θ angles. The observed XRD pattern indicated the following results:

(1) The peaks observed in the first region, especially at 20.26° and 23.5° angles, were detected in all samples; according to previous studies, these peaks belonged to PA6^45,46^. Gupta et al., besides mentioning the possible formation of two alpha and gamma crystalline phases in PA6, conducted phase detection by XRD analysis at different temperatures and observed similar peaks at 240 °C (similar to our results). These peaks were attributed to the dominance of the alpha phase over the gamma phase in PA6. They also observed the superiority of the alpha phase over the gamma phase in terms of thermodynamic stability^45^, which is a favorable feature of the composites investigated in the present study.

On the other hand, following phase extraction, the samples showed a red color in the graphs. After analysis and matching with the Joint Committee on Powder Diffraction Standards (JCPDS) cards, the Massicot cards (card No. 4747-338 and 05-0570) were also identified. According to previous studies, PbO is present in two crystalline phases of α-PbO and β-PbO^47^. While the alpha phase is tetragonal, the beta phase (i.e., Massicot) has an Orthorhombic shape with unequal sides. Also, in the graphs, the geometric dimensions of each sample are well characterized (a = 5.4, b = 4.7, c = 5.8 & α = β = γ = 90°).

(2) In addition to the abovementioned peaks, flattening of the primary regions (2θ angle < 10°) was also noticeable in the XRD graphs. This region is visible in Fig. 3A (crude PA6 sample). With an increase in the filler weight percentage, this area became smaller to the extent that it was hardly visible at the highest filler weight percentage (Fig. 3D). Meanwhile, enhanced peak intensities at other angles (2θ angle ≈ 20.26°, 23.5°, and 29.1°) could indicate more crystallization of the composites by increasing the weight percentage of lead (in exchange for a decrease in the amorphous region). Nevertheless, increasing the filler weight percentage did not induce tangible changes in the dominant shape or other parameters of the lattice.

### Thermal analysis of composites

**Figure 6:**
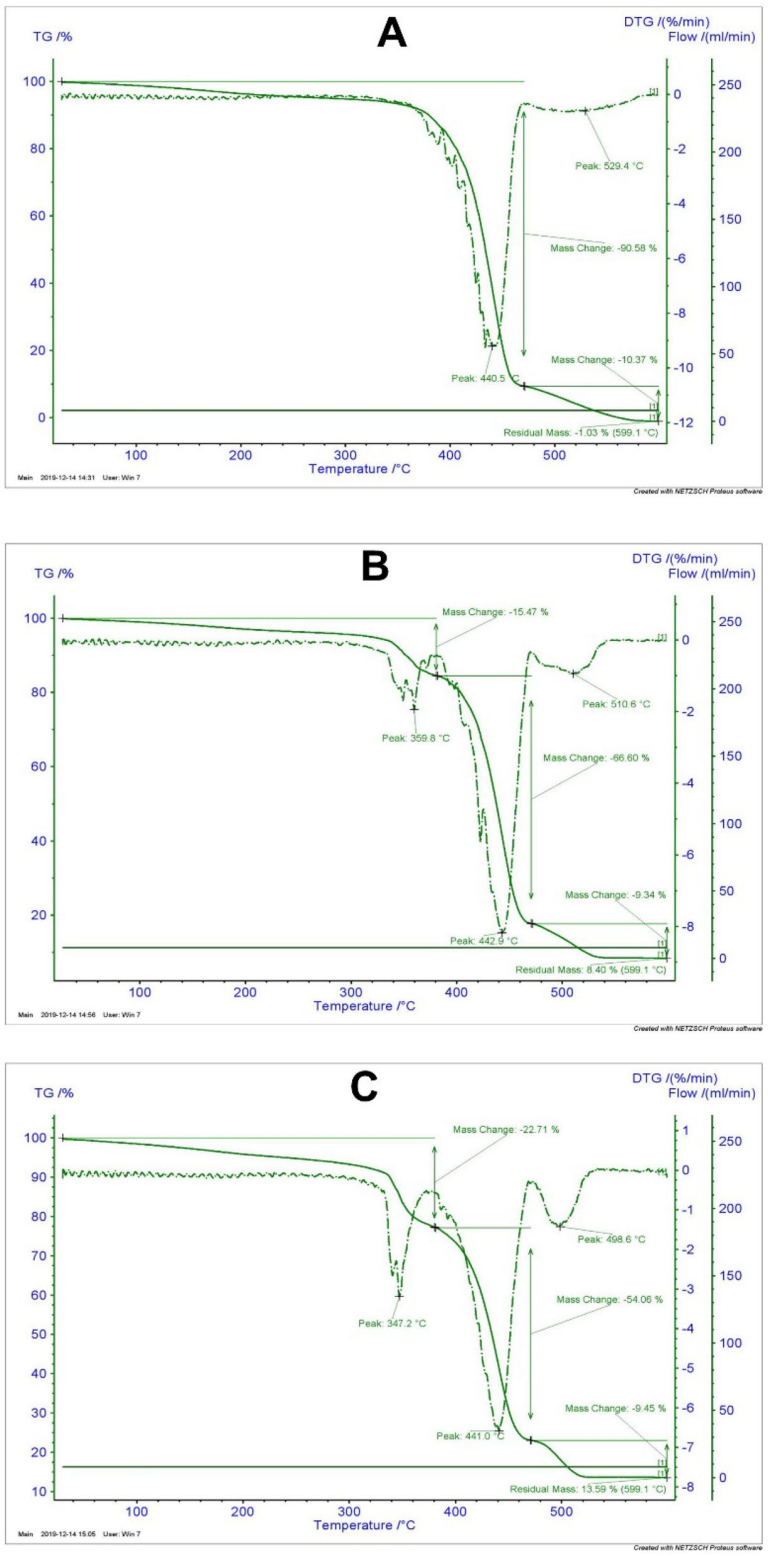
Analytical TGA and DTG graphs for (**A**) crude PA6, (**B**) PA6/PbO-10%, and (**C**) PA6/PbO-20%.

The thermal stability analysis of the three selected compounds, including crude PA6 and composites containing 10 and 20 wt.% PbO, was conducted, using a thermogravimetric analysis (TGA) and a differential thermal analysis (DTA); the results are presented in Fig. 6. The analyses were performed under N_2_ atmosphere in a temperature range of 20–600 °C (at a heating rate of 10 °C/min), according to the ASTM E1131-08 standard (2014).

The TGA curves revealed multi-phase trends in the mass changes of all composites, but at different intensities. For example, in the crude PA6 sample, the mass reduction was less than 10% before reaching a temperature of 400 °C, corresponding to a continuous glass transition in the DTA diagram; this trend is completely compatible with previous reports^48^. It should be noted that the mass change in this region was attributed to a reduction in moisture and dehumidification of the sample^49^. On the other hand, the mass change trends of samples containing 10 and 20 wt% lead were observed in the same area at a lower temperature, indicating a shift toward a lower glass transition temperature by increasing the filler weight percentage. The rates of mass reduction by increasing the lead concentration were 15.5% and 22.7%, respectively. In line with our observations, another study attributed this trend to the increased aggregate formation by the filler in the composite^10^.

Considering the trend of changes in the curves by increasing the temperature, the next round of decomposition occurred over a temperature range of 440–442 °C, where a sharp weight loss was clearly observed in all DTA graphs. The weight loss percentages compared to the initial peak were 90.5%, 66%, and 54% for the crude PA6, 10% lead-containing composite, and 20% lead-containing composite, respectively. The sharp changes in this area (known as the melting zone) can be attributed to chemical decomposition due to the loss of CO_2_ by the samples in this thermal range^49^. As can be seen in all three graphs, at the final temperature point (600 °C), the residual masses were 1%, 8%, and 13.5% for the crude PA6, 10% in the lead-containing composite, and 20% for lead-containing composite, respectively. Based on these observations, increasing the percentage of the lead filler led to a reduction in the weight loss and a boost in the thermal stability of the studied PA6/PbO composites.

### Results of gamma-rays attenuation on the samples

**Figure 7:**
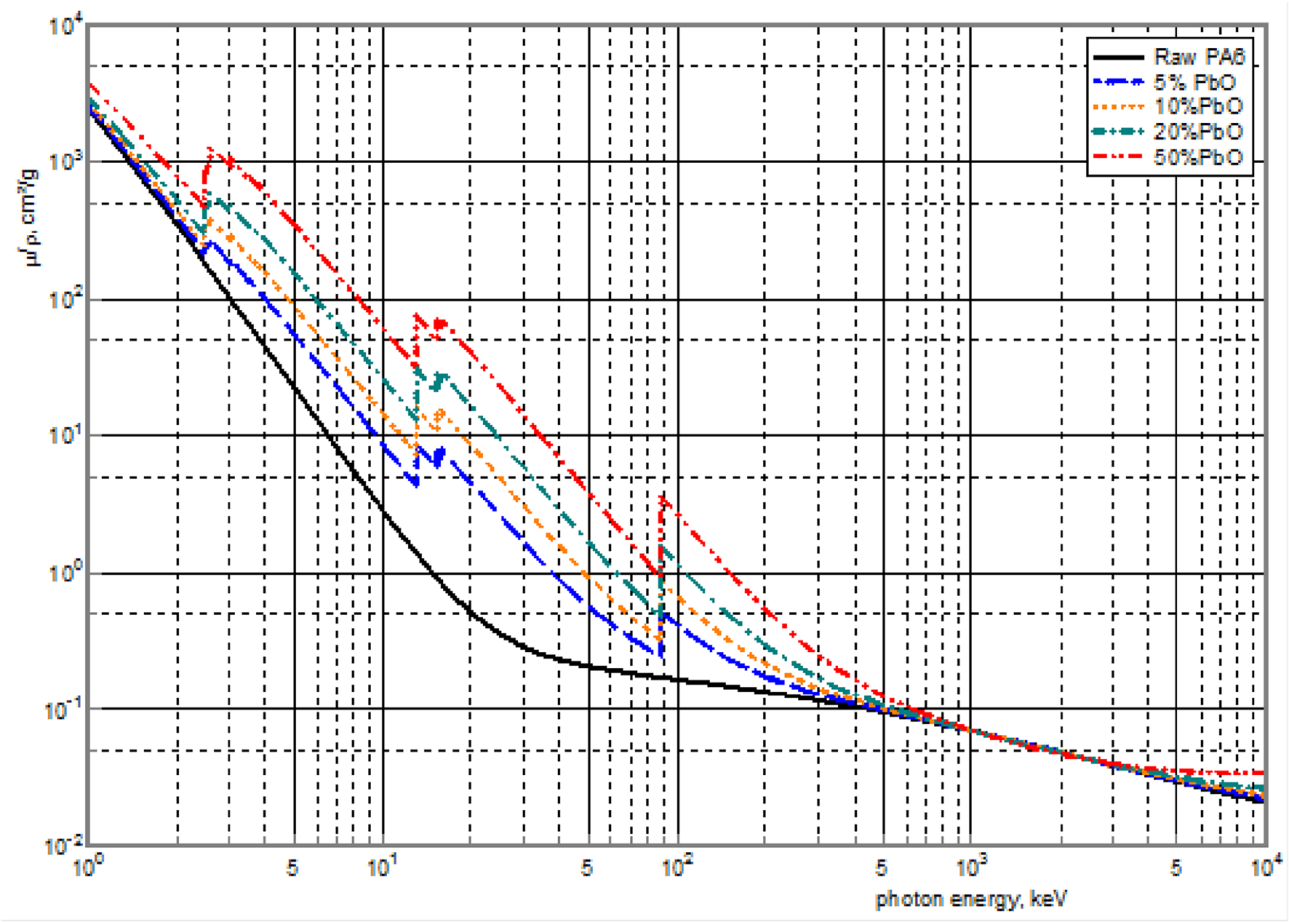
The trend of changes in the mass attenuation coefficients in a broad energy range for the five studied composites.

The mass attenuation coefficients (μ/ρ) of the five composites in question were calculated in the XMuDat. For a better understanding of this phenomenon, the trends were assessed in a photon energy range of 1–10,000 keV, as shown in Fig. 7.

As shown in Fig. 7, by increasing the energy level, the crude PA6 showed a slight gradual reduction in the attenuation coefficient. However, the stepwise addition of PbO up to 50% of the composite weight resulted in an increase in the attenuation coefficient, which is especially evident in the low- and medium-energy ranges (1–500 keV). Besides, the curve analysis of lead-containing composites showed a sudden noticeable drop in the mass attenuation coefficient in low-energy regions (< 100 keV) compared to higher-energy zones. The sharp peaks observed in this area might indicate the dominance of photoelectric effects at some energy levels (e.g., 88 keV and 15 keV for K- and L-absorption edges of lead, respectively)^6^. Based on these results, an increase in the weight percentage of lead offers a more optimal attenuation in the mentioned energy range.

Table 2 presents the linear attenuation coefficients calculated by the XMuDat program and the experimental method (μ_L_ ± ∆μ_L_) at two energy levels of 59 and 662 keV. Likewise, these results reported in previous studies^14–16^ with similar lead oxide filler percentages reported in columns 4 to 6 of this table. As can be seen, with increasing the filler weight percentage, the linear attenuation coefficients calculated through both methods in this study (columns 2 and 3) show a growing trend, indicating a good agreement with other works in the literature. However, with increasing the filler weight percentage, the differences between these results become more obvious. However, these differences in the standard deviations (± ∆μ_L_) of the calculated values (column 3) are also numerically noticeable.

**Table 2:** The linear attenuation coefficients obtained in the present study using the computational and experimental methods compared to the results of similar studies.

PbO wt%	Calculated by XMuDat (this study)	Experiments (this study)	Harish et al.^14^	Bagheri et al.^15^	Mahmoud et al.^16^
**E = 662 (keV)**					
5	0.102	0.101 ± 0.035	0.0997	0.089	-
10	0.109	0.091 ± 0.032	0.114	0.102	0.105
20	0.123	0.130 ± 0.046	0.1264	0.119	-
50	0.196	0.140 ± 0.049	0.206	-	0.189
**E = 59 (keV)**					
5	0.496	0.400 ± 0.140	-	-	-
10	0.800	0.605 ± 0.212	-	-	0.599
20	1.555	1.050 ± 0.369	-	-	-
50	5.130	4.050 ± 1.422	-	-	4.488

To better understand the reasons for these differences, we must first point out the possible reasons for the occurrence of uncertainty during the calculations. The possible reasons include random errors during counting by the detector system, as well as an increase in the dead time in the detector. Furthermore, geometrical errors and scattered photons that may have been detected can cause the geometry used to deviate from an ideal parallel narrow geometry, and consequently deviate the measured values from the expected ones^50,51^. The last point is especially applicable at lower energies (e.g. 59 keV in this study). However, the reasons for the differences observed in the higher filler weight percentages (here from 20% by weight upwards) can mostly be attributed to the asymmetric distributions of the lead oxide fillers in the polyamide. In other words, the XMuDAt program assumes that the elements are uniformly distributed in the samples, but in practice, as seen in the SEM images (Fig. 3), this distribution was not necessarily symmetric, and as the filler percentage increased, more agglomerates were also observed. Therefore, this can reduce the linear attenuation parameter (and cause less attenuation) in practice as highlighted in some studies^10,52^.

Moreover, a comparison of the data from this study with the findings reported in previous studies confirms our observations (increasing the attenuation coefficients with increasing the filler concentrations) but also reveals some differences. It should be noted that despite the similarity in the percentage of lead oxide filler, the difference in the type of polymer matrix can be also highlighted. For example, Harish et al.^14^ used unsaturated polyester (1.2 g/cm^3^); Bagheri et al.^15^ used unsaturated polyester plus 5% nanoclay filler; and Mahmoud et al.^16^ used a high-density polyethylene (0.953 g/cm^3^). These differences caused slight variations in the amount and type of elements in the products and consequently led to variable degrees of attenuation. As the final density of the product seems to affect the radiation attenuation^15^, accurate measurement of the final density of materials can somehow explain the observed differences.

**Figure 8:**
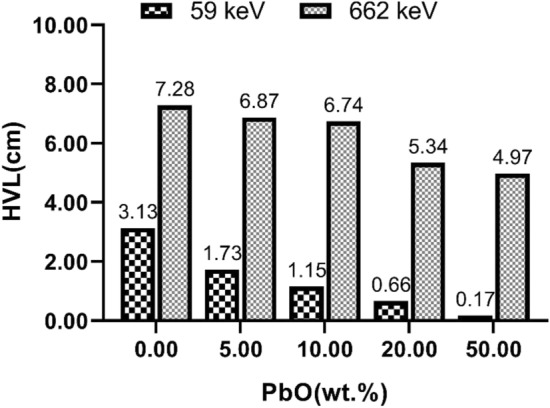
The measured HVL values for the five composites studied at energy intensities of 59 and 662 keV.

Finally, the gamma radiation attenuation improved by increasing the weight percentage of the lead oxide, as evidenced by the measured HVL values (**Fig. ****8**). By increasing the weight percentage of the lead oxide from 0% (crude polyamide) to 50%, the HVL value decreased from 3.13 to 0.17 cm at an energy intensity of 59 keV and from 7.28 to 4.97 cm at an energy intensity of 662 keV. In other words, by increasing the energy and penetrating depth of gamma rays, the thickness required to block half of the irradiated gamma rays also increased.

### Mechanism of radiation interactions with lead composites

**Figure 9:**
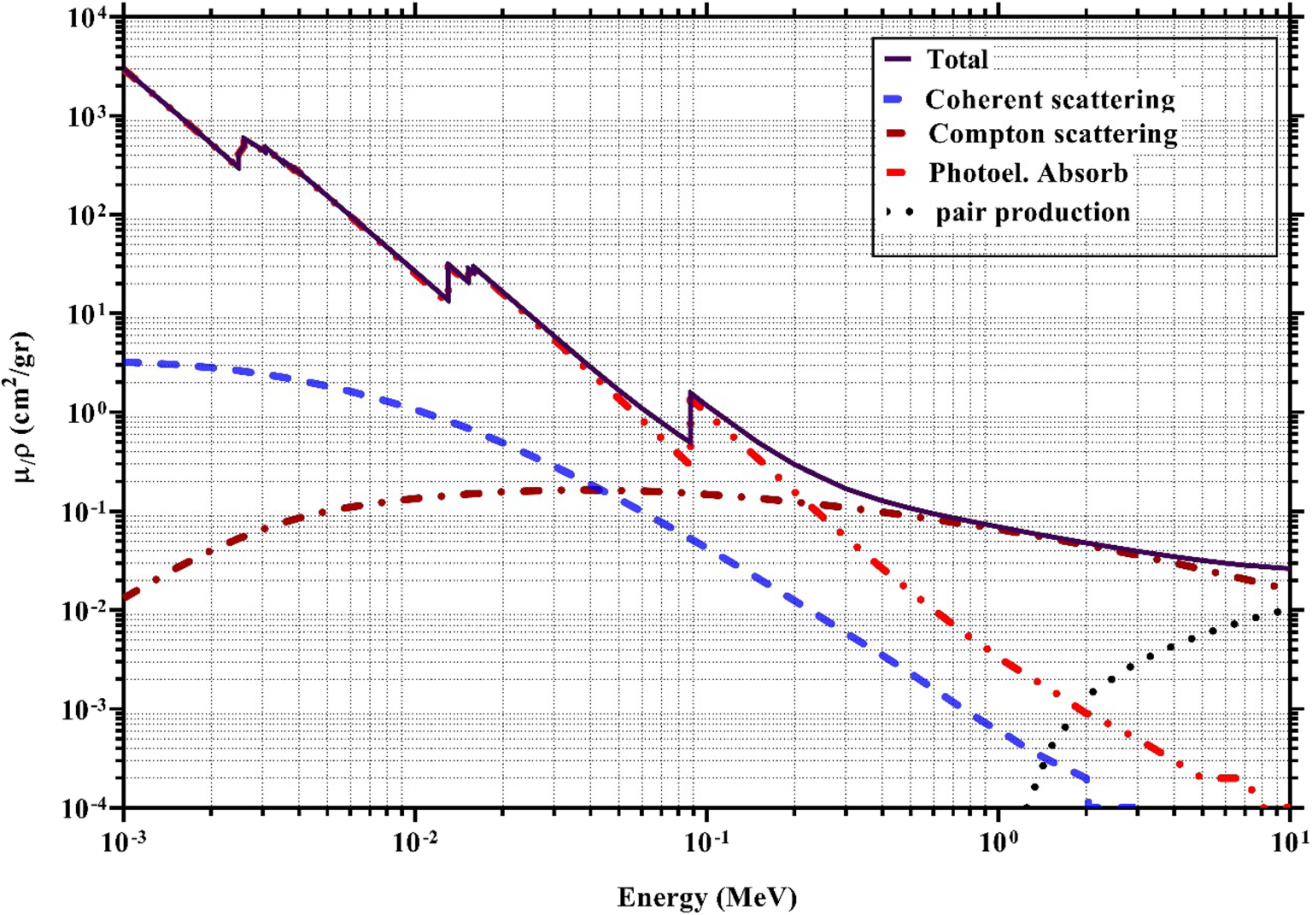
The partial and total mass attenuation coefficients for the selected sample (PA-PbO-20%).

To understand the shielding effect of composites against ionizing photons (X- and gamma rays), previous studies have addressed possible interactions of photons colliding with shielding materials^53,54^. To illustrate the shielding mechanism, one of the samples containing lead filler (PA6/PbO-20%) was selected in the present study, and its possible interactions were investigated by exhibiting the partial (including coherent, Compton, photoelectric, and pair production) and total mass attenuation coefficients in the selected energy range energies (0.001–10 MeV)^39^, and the outcomes are plotted in Fig. 9.

As shown in Fig. 9, in a low energy range, the predominant interaction was the photoelectric absorption effect. This effect is well observed up to the energy level of 0.2 MeV (where the two brown and red curves intersect). However, in the same energy range, the coherent dispersion and the Compton effect are changing differently, so that the former is constantly decreasing and the latter (the Compton effect) is increasing. It should be noted that the energy transformation to the medium is almost negligible in the coherent event^55^.

The sharp peaks seen in some energy levels (e.g. 0.015 and 0.088 MeV) are well compatible with the L and K adsorption edges of lead^6^, promising high absorption efficiencies with the intensified photoelectric effect. As the energy level increases, the Compton effect is gaining momentum, and this trend continues until the energy reaches its maximum level (10 MeV). At the same time, a probable decline in the photoelectric effect in its curve is expected. Finally, we first observe the emergence of the pair production effect (at the threshold energy of 1.22 MeV) and then its continuous increase. Following this observation, the first region of low-energy photons can be referred to the absorption region (due to the predominance of the photoelectric effect), and the higher energy regions can be referred to the attenuation region (due to the increased probability of scattering phenomena with adsorption).

## Discussion

In recent years, due to the increasing demand of ionizing radiation in various industries and medicine, the importance of protection against radiation has increased^1,2^. In this regard, polymeric composites, as novel alternatives to traditional materials, have been proposed for designing protective shields and addressing the challenges of conventional methods^6,8,13^. Previously, polymeric composites were used successfully for the fabrication and design of radiation dosimeters^23–30^.

The disadvantages of using bulk amounts of lead for constructing radiation protectants include high toxicity, environmental pollution, undesirable mechanical stability, and high weight of lead products (due to the high lead density). However, due to its high atomic number, wide availability, and most importantly, its capacity to transform into micro- and nano-dimensions (in various chemical forms) promise the optimized reuse of lead, which has shown satisfactory results in the previous studies^6–8^.

In the present study, new PbO/PA6-based composites with different weight percentages of PbO (0, 5, 10, 20, and 50%) were fabricated and then characterized using both computational (XMuDat program) and experimental methods (characterization and experimental tests). According to the computational analyses (Fig. 1), before the synthesis of composites, lead-containing samples were predicted to show better attenuation compared to lead-free samples (crude PA6). By increasing the weight percentage of PbO, the attenuation trend improved, as evidenced by an elevation in the mass attenuation coefficients. This finding was more evident (as peaks) at energy intensities of 15 and 88 keV, which represented the K- and L-absorption edges of lead and indicated the predominance of the photoelectric effect in these composites^6^. It seems that in diagnostic procedures, such as radiology and nuclear medicine, these composites can be effective in protection against radiation.

In the experimental section of this study, the composites were initially fabricated and then characterized. For this purpose, the melt-mixing method, which is a cost-effective and relatively convenient method, was used to synthesize the samples in an extruder. The required adjustments in the device, such as temperature (based on the melting point of PA6) and the rotation speed and duration, were set based on the operator’s experience and the results reported in a study by Mahmoud and colleagues^16^. The experiments were conducted in a specialized laboratory for fabricating plastics and polymers.

The characterization of the samples based on SEM showed that up to a filler weight percentage of 20 wt%, PbO particles were distributed almost symmetrically in the polyamide. However, by increasing the weight percentage, the filler started to form aggregations seen as large lumps at the highest weight percentage (50%). The EDX analysis of a selected sample revealed the fingerprints of lead and several other elements in the composites (Fig. 4). To explain the increasing accumulation of filler particles, Mehrara et al*.* referred to the formation of strong van der Waals forces among filler particles, dominating the filler–polymer bonds^10^. This observation was consistent with previous reports on other lead-containing composites with similar weight percentages^16^. It should be noted that in this study, for the first time, an SEM image of a sample containing 50% lead oxide was acquired.

The phase-detection and evaluation of the crystal network of the composites via XRD showed that PA6 appeared in the form of alpha crystals in the samples. Previous studies have reported that this type of polyamide is sensitive to temperature and may form different phases upon thermal changes^45,48^. In this study, the emergence of peaks at 20.26° and 23.5° angles indicated the predominance of the alpha phase, which might be attributed to the sample synthesis and pressing processes at temperatures approximating 240 °C. Besides, by increasing the filler weight percentage, the flattening of initial zones (2θ angle < 10°; amorphous zone) started to decrease, whereas greater sharpness and more crystallization were observed at angles corresponding to PbO. This finding was also confirmed after matching with the JCPDS standard cards and detection of the Massicot phase (lead mineral form) (codes 4747–1387 and 05–0570, respectively) (Fig. 5). These observations were in agreement with previous studies^16^.

In the current study, thermal stability was assessed using the TGA and DTGA analyses in three samples (crude PA6 and composites containing 10% and 20% lead oxide, respectively). In the crude PA6 sample (Fig. 6A), before reaching a temperature of 400 °C, there was a relatively smooth and continuous glass transition, which could be attributed to the dehumidification of the sample^49^. It should be noted that for the samples containing a lead oxide filler (Fig. 6B,C), the glass transition temperatures were lower (359 °C and 347 °C for 10% and 20% filler weight percentages, respectively). A similar trend was also observed in a previous study^10^, which could be attributed to the presence of lumps and deformations in the mentioned temperature range. By increasing the temperature and reaching the melting zone, the superiority of lead-containing samples was supported by their less significant weight loss. This superiority remained noticeable until the end-point temperature (600 °C) and was verified by the quantitative assessment of the residual mass. Therefore, it can be concluded that increasing the lead filler ratio in the composites caused them to lose less weight and acquire higher thermal stability.

In the second section of the experimental analyses, the radiation attenuation trends for photon sources were assessed at 59 keV (low energy) and 660 keV (medium energy) in the lead-containing samples in a specialized laboratory. The experimental results were consistent with the theoretical data for lower filler weight percentages. To explain this finding, it should be noted that in the computational method, it is assumed that the samples have a homogeneous distribution of elements. Thus, in practice, the samples with less formation of lumps showed a better agreement with the computational data. On the other hand, significant differences were observed in the samples with a higher filler percentage (e.g., 50%) due to the formation of numerous lumps.

Likewise, Mahmoud et al. examined lead filler weight percentages up to 10% and reported a good agreement between the experimental and computational data due to the uniform distribution of the filler^56^. The experimental data in the present study indicated by increasing the weight percentage of lead from 0% (crude PA6) to 50%, the HVL values decreased from 2.13 to 0.17 cm at an energy level of 59 kV and from 7.21 to 4.97 cm at an energy level of 662 keV.

This study was conducted with some limitations. Thus, we provided some suggestions for future studies. Considering the efficiency of the composites containing lead up to a weight percentage of 20%, composites with other lead weight percentages (20% to 50%) can be assessed in future experiments. In this study, thermal stability was investigated in only three samples; it is advisable to extend this analysis to other samples, as well. Finally, further experimental analysis of the radiation attenuation and absorption capacity of PbO/PA6 composites can be performed at other energy levels to verify the protective efficiency of these composites. To the best of our knowledge, this study on PA6/PbO composites is among the first reports on these novel radioprotectants; therefore, further investigation is strongly recommended.

## Conclusion

This study explored the applicability of novel PA6/PbO-based composites, containing different weight percentages of PbO (0, 5, 10, 20, and 50%) as protectants against X-ray and gamma rays, using both computational and experimental methods. The composites were synthesized in a laboratory mixing extruder and characterized by various tests, including SEM, XRD, TGA, and experimental radiation tests. The SEM images showed the acceptable symmetrical distribution of PbO particles (weight percentages up to 20%) in PA6. Moreover, an increase in the formation of crystalline networks, along with decreased amorphous zones in the composites, was observed by increasing the filler weight percentage, as evidenced by the XRD analysis. Besides, a thermal analysis was conducted for the selected samples, containing 0%, 10%, and 20% PbO from room temperature up to 600 °C. As shown in the TGA and DTGA analyses, the total weight loss reduced, and thermal stability improved in the lead-containing composites. However, a descending trend in the glass transition temperature was seen in the samples containing fillers, which was probably related to aggregates deformation.

Overall, at higher energies for lower reinforcement phase loadings, the experimental results were consistent with the computational data. Probably, agglomeration of heavy metal oxide nanoparticles at higher concentrations led to a decrease in the linear attenuation coefficients.

However, the formation of lumps and heterogeneity in the filler distribution of the composite could markedly affect the radiation protection efficiency. The data showed that a higher heterogeneity would result in a greater deviation of the measured attenuation coefficient from the calculated value. Here, at the highest filler weight percentage (50%), this heterogeneity reduced the consistency between the experimental and computational data and reduced the radiation attenuation efficiency. Since PA6/PbO-based radioprotectant composites are novel materials, it is strongly recommended to further investigate their characteristics in future experiments while considering the limitations of this study.
